# Wound Chemotherapy by the Use of Negative Pressure Wound Therapy and Infusion

**Published:** 2010-01-08

**Authors:** Nicholas A. Giovinco, Trung D. Bui, Timothy Fisher, Joseph L. Mills, David G. Armstrong

**Affiliations:** Southern Arizona Limb Salvage Alliance (S.A.L.S.A.), University of Arizona College of Medicine, 1501 N Campbell Ave, Tucson, AZ 85724

## Abstract

**Introduction:** Although the use of negative pressure wound therapy (NPWT) is broadly efficacious, it may foster some potentially adverse complications. This is particularly true in patients with diabetes who have a wound colonized with aerobic organisms. Traditional antiseptics have been proven useful to combat such bacteria but require removal of some NPWT devices to be effective. **Methods:** In this article, we describe a method of “wound chemotherapy” by combining NPWT and a continuous infusion of Dakins' 0.5% solution either as a standardized technique in one device (ITI Sved) or as a modification of standard technique in another (KCI VAC) NPWT device. The twin goals of both techniques are to effectively reduce bacterial burden and to promote progressive wound healing. **Results:** We present several representative case examples of our provisional experience with continuous streaming therapy through 2 foam-based negative pressure devices. **Discussion:** Wound chemotherapy was successfully applied to patients with diabetes, without adverse reactions, complications, or recolonization during the course of treatment. We believe this to be a promising method to derive the benefits of NPWT without the frequent adverse sequela of wound colonization.

Negative pressure wound therapy (NPWT) is frequently employed to achieve wound simplification and closure in patients with diabetes.^1^ The application of a vacuum device is simple and automatically maintains a negative pressure environment, conducive for wound healing.^2^,^3^

One of the problems we have noted in our extensive experience with NPWT in very complex and chronic wounds has been the development of wound colonization and subsequent maceration. When used for extended periods, such a bacterial burden could necessitate VAC device removal and discontinuation of NPWT.^4^ Although in vitro studies of silver-impregnated foam dressings have shown promise, further evidence from in vivo trials is needed.^5^ In our previous experience with such dressings, we have not managed to reduce bacterial burden to our satisfaction.

Previous authors, most notably Fleischmann and colleagues, as well as Svedman, have discussed the infusion of a variety of substances into the wound.^6^^-^12 The work of Fleischmann led to the development of a “VAC Instill” device, which consists of a VAC port and an infusion port with 2 “stopcocks” to allow for separate infusion and vacuum periods. This process involves a “hold” cycle during which fluid is infused into the foam during a pause in NPWT.

On the basis of our experience, the use of the latter device poses 3 difficulties: (1) VAC Instill therapy system has not been readily or rapidly available to many units; (2) the programmed “hold sequence” often potentiates maceration of the wound and its margins; and (3) the quality of dressing seal appears to be compromised by stagnant irrigation.

The Svedman concept involves continuous infusion, or “streaming,” with simultaneous NPWT and is realized in a commercially available device (Sved; Innovative Therapies, Inc [ITI], Gaithersburg, Md). This streaming concept is attractive for the aforementioned reasons. The ITI Sved unit works by means of a constant vacuum drainage mechanism, whereby an infusion or instillation catheter is connected to a secondary port in the foam dressing. Because both an infusion of therapeutic agents and a significant reduction in wound margin macerations can be achieved without compromise of dressing seal integrity, the Sved device has shown some promising preliminary results. However, access to this device has been difficult in our unit and others.

To address these issues we have identified with the standard VAC and the VAC Instill as well as the lack of availability of the ITI Sved device and modified the KCI VAC device to permit continuous streaming infusion using equipment found in any healthcare setting. In addition to the standard drainage port, a secondary port is installed on the opposite side of the vacuum tubing to insert an intravenous (IV) infusion catheter. This interface is then sealed with additional dressing over the foam.

In the following cases, we describe the use of ITI Sved and modified KCI VAC devices, with a chemotherapeutic 0.5% Dakins' (dilute hypochlorous acid) solution, to achieve wound healing and closure.^13^^-^15 Foam dressings were reapplied every other day with an infusion rate of approximately 30 mL/h. Debridement of wound margins, base, and exposed bone were performed as necessary (Fig 1).

## CASE STUDIES

### Case 1

A 60-year-old man with diabetes mellitus presented with an infection caused primarily by methicillin-resistant *Staphylococcus aureus* and underwent an open-Chopart amputation of his right foot. While on KCI VAC therapy, he developed heavy wound colonization with *Pseudomonas aeruginosa*.

After a series of surgical debridements and 2 autologous split-thickness skin grafts, Sved device therapy was initiated with an infusion of Dakins' solution. Substitution of a KCI VAC was utilized to allow for a larger collection canister while maintaining the ITI Sved infusion catheter.

Reinfection from *Pseudomonas* or any other pathogens did not occur while Dakins' solution as an infusion medium was administered from a modified KCI VAC or Sved over the span of 26 days until the application of the ultimate split-thickness skin graft.

### Case 2

A 40-year-old woman with diabetes mellitus presented following a Chopart-level amputation and failed free flap of her left foot. While undergoing KCI VAC therapy, she developed a colonized wound; wound cultures grew *Pseudomonas*. This colonization and periwound maceration necessitated discontinuation of the VAC device.

After multiple surgical debridements and drainage procedures, the application of a modified KCI VAC device and dressing with a Dakins' IV solution as an infusion medium via IV pump was performed. While receiving Dakins' solution as an infusion medium with NPWT over a 16-day follow-up course that included 5 days in hospital and 11 days in home therapy, reinfection with *Pseudomonas* did not reoccur. The wound was prepared for split-thickness skin grafting and healing by secondary intention.

### Case 3

A 65-year-old man with diabetes and a history of left above-knee amputation was seen in the outpatient clinic with an ischemic ulcer over his right medial malleolus. He had previously undergone a cryovein (cadaveric vein conduit) bypass and multiple free flaps without successful healing or wound closure.

Previous therapy with conventional NPWT resulted in wound colonization with *Pseudomonas*, necessitating discontinuation of the VAC. After a series of surgical debridements, an ITI Sved device with Dakins' solution as an infusion medium was applied. This patient was monitored over the course of 16 days (3 days as inpatient and 13 days at home). *Pseudomonas* colonization did not recur while NPWT with Dakins' solution as an infusion medium was maintained. His wound was well prepared for a subsequent split-thickness skin graft.

### Case 4

A 56-year-old man with diabetes and delayed healing of a transmetatarsal amputation underwent multiple revascularization and debidement procedures. Previous application of an unmodified KCI VAC therapy device resulted in colonization and eventual *Pseudomonas* infection. Surgical debridement was performed, followed by the application of a modified KCI VAC dressing with an IV infusion pump.

During the time of infusion with NPWT via Dakins' solution, his wound did not become reinfected or suffer any further with ischemic necrosis. The patient's wound was well prepared for a subsequently successful split-thickness skin graft.

## OUTCOMES

**Figure 2:**
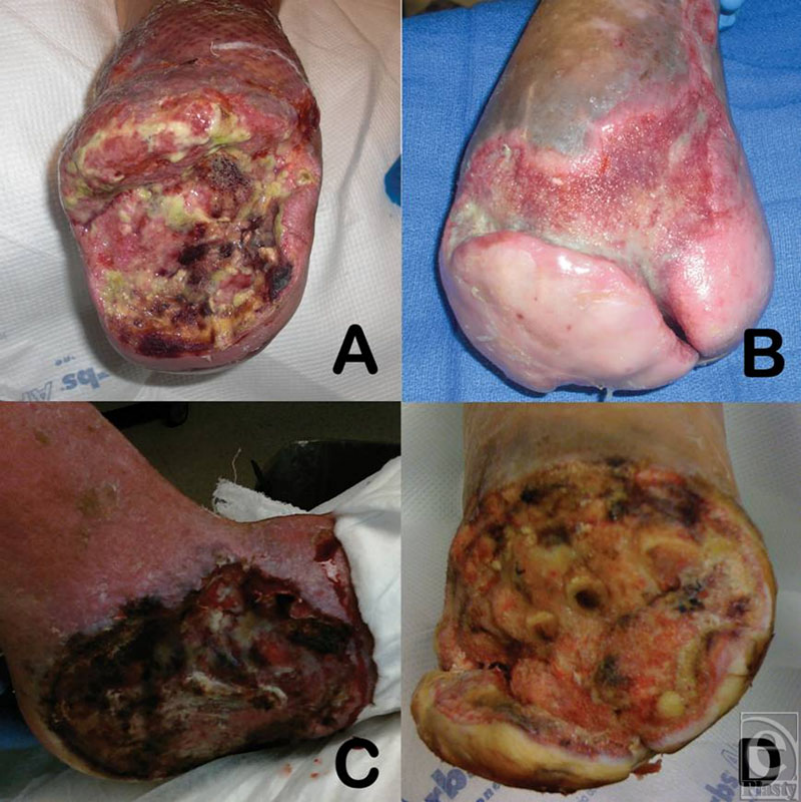
Examples of cases 1 to 4 before wound chemotherapy treatment: (*a*) case 1; (*b*) case 2; (*c*) case 3; and (*d*) case 4.

**Figure 3:**
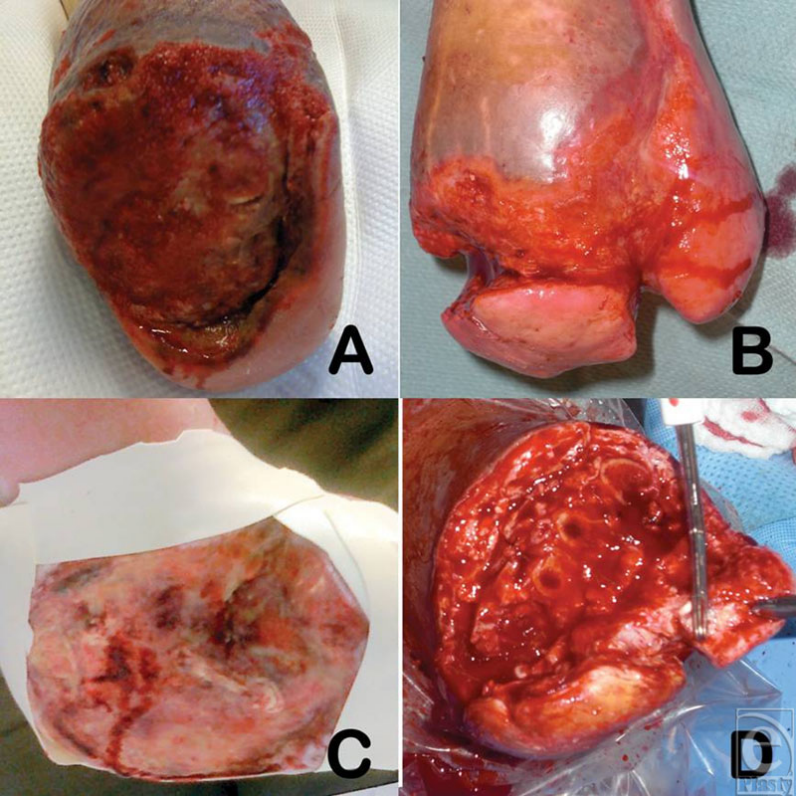
Examples of cases 1 to 4 after wound chemotherapy treatment for the number of followed days: (*a*) case 1; (*b*) case 2; (*c*) case 3; and (*d*) case 4.

All 4 patients suffered from chronic, complex, diabetic extremity wounds, previous amputations, and failed standard NPWT therapy. NPWT failure was due to colonization and slough replete with *Pseudomonas* species and required discontinuation of therapy. When NPWT treatment combined with a continuous infusion of Dakins' 0.5% solution was initiated, reinfection or recolonization of the wounds did not occur (Figs 2 and 3).

**Figure 4:**
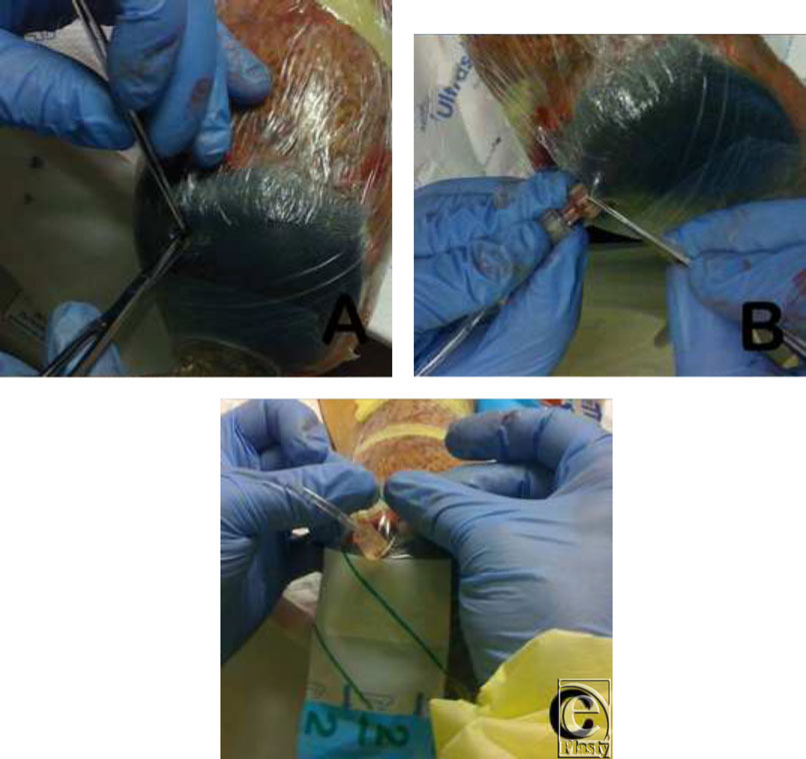
A 3-step method for the modification of a standard VAC foam dressing with a standard disposable suture removal kit. (*a*) Step 1: The initial slit in the dressing using scissors and countersinking of underlying foam using tweezers to accommodate the interface of the intravenous (IV) tubing. (*b*) Step 2: Insertion of the IV tubing interface into the foam through the covering dressing. (*c*) Step 3: The application of dressing seal around the IV tubing.

Surgical debridements were performed as necessary during dressing changes at the bedside or in the operating suite. The combined use of Dakins' solution as an infusion medium and NPWT did not appear to adversely impact wound healing rates; in fact, they appeared to be accentuated during the period of evaluation (Fig 4).

## DISCUSSION

The use of NPWT alone may, in some patients, promote a change in the wound microbiota and result in the colonization that can impede healing. The cyclical instillation, hold, and drainage of therapeutic fluids with a “VAC Instill” device pose several problems including wound maceration and loss of dressing seal. The use of streaming Dakins' solution as an infusion medium appears to resolve many of these problems. We believe that the possibilities for streaming therapy through NPWT to be broad and not simply limited to antimicrobials. Modalities such as analgesics, cytokines, angiogenic factors, and anti-inflammatory agents may also prove fruitful in the future.

To our knowledge, the combination or modification of vacuum devices to permit continuous Dakins' solution as an infusion medium has not been previously published. Although further research is necessary to ascertain the true value of Dakins' solution as an infusion medium or any such infusion into NPWT devices, we believe wound-based chemotherapy should be considered for a place in the treatment spectrum of complex wounds. The possibility of standardizing and streamlining the delivery of targeted therapies is not only attractive but also necessary as we move toward more controlled therapeutic regimens. We anticipate further developments in this area.

## Figures and Tables

**Figure 1:**
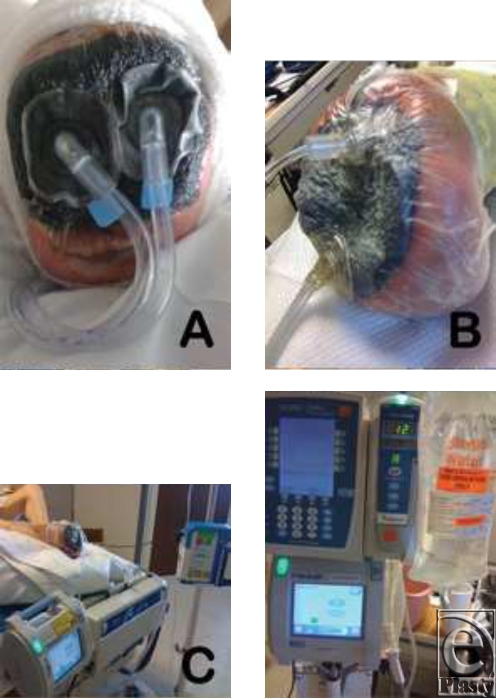
Four images to exemplify the devices, dressings, and modifications used in this study. (*a*) ITI Sved foam dressing with an infusion and drainage port; (*b*) a modified KCI VAC dressing with an intravenous (IV) pump infusion catheter; (*c*) KCI VAC with Sved infusion catheter; and (*d*) a modified KCI VAC foam dressing with an IV pump infusion catheter.
